# Teaching styles and sports engagement: mediation by satisfaction and resilience in Chinese adolescents

**DOI:** 10.3389/fpsyg.2025.1630300

**Published:** 2025-07-07

**Authors:** Liepeng Huang, Fengjuan Tian, Furong Li

**Affiliations:** ^1^Faculty of Education, Shaanxi Normal University, Xi'an, Shaanxi, China; ^2^Shaanxi Vocational and Technical College, Xi'an, Shaanxi, China

**Keywords:** physical education teacher teaching styles, classroom satisfaction, psychological resilience, sports learning engagement, mediation analysis

## Abstract

**Purpose:**

This study examined the associations between perceived PE teacher teaching styles on Chinese middle school students' sports learning engagement, and the mediating roles of classroom satisfaction and psychological resilience.

**Methods:**

A cross-sectional survey assessed 1,629 junior and senior high school students in Chengdu and Chongqing, Validated scales measured perceived teaching styles, psychological resilience, sports learning engagement, and PE class satisfaction. SPSS 26.0 was used for correlation, regression, and mediation analyses (SPSS PROCESS v4.1 Macro, Model 4).

**Results:**

Perceived teaching styles were positively associated with sports learning engagement (β = 0.371, 95%CI = 0.343–0.398, *p* < 0.01). Significant associations emerged for rigorous-logical (β = 0.292, 95%CI = 0.066–0.518, *p* < 0.05), caring-sharing (β = 0.489, 95%CI = 0.179–0.800, *p* < 0.01), and innovative-exploratory (β = 0.898, 95% CI = 0.523–1.273, *p* < 0.01) styles, but not for humorous-active (β = 0.095, 95% CI = −0.092–0.282, *p* > 0.05).Classroom satisfaction and psychological resilience partially mediated the relationship between teaching style and learning engagement (*p* < 0.05), with the mediating role of psychological resilience (26.0%) being significantly stronger than that of classroom satisfaction (18.9%).

**Conclusion:**

There is a significant positive association between PE teachers‘ teaching styles and students' PE learning engagement. Moreover, classroom satisfaction and psychological resilience play mediating roles in this relationship. Therefore, it is recommended that PE teachers prioritize the adoption of positive teaching styles in PE classroom instruction.

## 1 Introduction

Learning engagement is a multidimensional positive psychological state that involves the profound integration of cognitive, affective, and behavioral dimensions (Fredricks et al., 2004; Skinner et al., 2009). In the realm of physical education, sports learning engagement is defined as an individual's active engagement and sustained endeavor to master movement knowledge and skills, in addition to a clear preference for physical activities and positive emotional experiences (Bevans et al., 2010). Sports learning engagement reflects the dynamic integration of physical activity and psychological state (Chen and Hypnar, 2015). In particular, it is demonstrated in three dimensions: (1) Vigor engagement (e.g., proactive reflection on movement strategies); (2)Dedication engagement (e.g., interest and enjoyment in physical activities); (3) Absorption engagement (e.g., persistent practice of motor skills) (Owen et al., 2016). It is important to note that sports learning engagement is not only directly linked to improvements in the physical health of adolescents (Rubinelli et al., 2019), but it also improves social adaptability through physiological mechanisms such as endorphin release and anxiety reduction during participation (Lubans et al., 2016). It also reduces academic tension (Diamond and Ling, 2016), reduces the risk of academic burnout (Ye et al., 2024), and fosters mental health development (Langford et al., 2014). The significance of engaging young individuals in sports education for their overall growth and development is underscored by the discovery. Consequently, researchers are currently concentrating on methods to enhance student engagement in sports-related learning.

Since the 1970s, educational research has been deeply concerned with the concept of teaching style, which serves as a fundamental representation of the instructional behaviors of instructors (Biggs, 2001). “Teaching style” is broadly defined by scholars as the consistent behavioral preferences of teachers in the areas of problem-solving, task implementation, and interactions (Brown, 2025; Sternberg, 1997), This includes the use of voice, gestures, and movements to elicit emotional responses or capture and maintain students' attention (Gafoor and Babu, 2012). Autonomy-supportive styles, which are guided by self-determination theory (Ryan and Deci, 2017), are linked to higher intrinsic motivation and behavioral engagement (Leenknecht et al., 2017). Structured styles, on the other hand, reduce uncertainty in skill learning to strengthen cognitive engagement (Martin, 2008). Emotionally supportive styles foster affective engagement by fulfilling belongingness needs (Shen et al., 2024). Empirical evidence supports teaching style as a key correlate of engagement in sports learning (Yang and Dong, 2017). Nevertheless, the heterogeneous associations of teaching strategies may be a result of cultural differences between Eastern and Western contexts (Sun et al., 2017). He et al. (2011) proposed four associationive teaching styles—humorous-active, rigorous-logical, caring-sharing, and innovative-exploratory—that were specifically designed for the dynamics of Chinese classrooms. Humorous-active (HA) physical education teachers can associationively capture students' attention and increase their participation in class by creating a lively classroom atmosphere (Ashwin et al., 2020). Rigorous-logical (RL) physical education teachers promote systematic mastery of sports knowledge and skills through structured content, inspiring focused engagement (Akram and Li, 2024). Caring-sharing (CS) physical education teachers strengthen teacher-student trust by addressing students' emotional needs and individual differences, inspiring emotional engagement (Biggs et al., 2022). Innovative-exploratory (IE) physical education teachers stimulate curiosity and proactive exploration through creative teaching activities, inspiring energetic engagement (Chow et al., 2021). These styles provide a localized framework for the analysis of sports learning engagement. Due to current research has primarily concentrated on cognitive disciplines such as mathematics (Opdenakker and Van Damme, 2006), English (Ghanizadeh and Jahedizadeh, 2016), and physics (Ridwan et al., 2019), it fails to consider distinctive factors in physical education, including physical exertion and risk perception (Goudas et al., 1994). Consequently, it is imperative to examine the associations between students' perceptions of PE teaching styles and their engagement in sports learning in Chinese contexts in order to advance the field of physical education pedagogy. The study proposes the following hypothesis in light of this literature review:

H1: The perceived PE teaching styles (rigorous-logical, humorous-active, caring-sharing, innovative-exploratory) among middle school students are significantly associated with their PE learning engagement.

Classroom satisfaction is the comprehensive assessment of the learning outcomes, instructional content, teacher-student interactions, and classroom environments by students (Alqasa and Afaneh, 2022; Guolla, 1999). Classroom satisfaction in physical education is contingent upon the instructional styles of PE instructors (Betoret, 2007). Studies suggest that motivational and supportive teaching styles increase students' motivation to participate in extracurricular physical activities by promoting positive psychological and physical experiences (Diloy-Peña et al., 2021). Consequently, classroom satisfaction is improved (Betoret, 2007; Chatzipanteli et al., 2015). Additional research indicates that classroom satisfaction serves a dual function in the context of sports learning: Cognitive and behavioral engagement are directly stimulated by high satisfaction (Moore and Fry, 2017). Conversely, low satisfaction may result in a decrease in students' participation in physical activities (Baños et al., 2020). This connection between instructors and students in the classroom setting suggests that the level of satisfaction experienced by students is a significant factor in the interaction between teaching methods and student engagement in learning activities. Based on this observation, the research proposes a hypothesis.

H2: The relationship between the teaching styles of PE instructors and the engagement of students in sports learning is mediated by classroom satisfaction.

Psychological resilience is the quality of an individual's ability to adapt, recuperate, and maintain psychological functioning in the presence of adversity. In addition to alleviating emotional distress, resilience also enhances the abilities of adolescents, promoting the development of coping strategies to confront challenging situations (Liebenberg and Scherman, 2021). In the context of sports learning, psychological resilience is particularly important: Coping with adversities: It allows adolescents to associationively manage negative factors (e.g., sports injuries), task-related challenges (e.g., complex motor skills), and peer-related pressures in PE settings. Resilience, which regulates stressors, improves the persistence and enthusiasm of sports learning engagement (Biggs et al., 2024; Trigueros and García-Mas, 2025). Maintaining Motivation: Emotional regulation and self-efficacy are enhanced by resilience (Cetinkalp and Turksoy, 2011), which in turn mitigates negative emotions precipitated by failure or setbacks, thereby preserving intrinsic motivation for sustained sports participation (Biggs et al., 2024). Although the beneficial associations of resilience on adolescent mental health are well-documented (Mesman et al., 2021), the specific pathways by which it links PE teaching strategies to sports learning engagement are still unexplored. Based on this synthesis, the subsequent hypothesis is proposed in the study:

H3: The relationship between the teaching styles of physical education instructors and the involvement of students in sports learning is mediated by psychological resilience.

Consequently, prior research has investigated instructional methodologies and learner engagement; however, limited studies have analyzed the mediation associations of psychological resilience and classroom satisfaction within sports contexts, especially among Chinese teenagers. This study seeks to elucidate the associations between students' perceptions of physical education teachers' pedagogical styles on their engagement in physical education within Chinese classrooms while also investigating the mediating roles of classroom satisfaction and psychological resilience in this relationship. This study aims to enhance the comprehension of the mechanisms governing teaching styles and physical education learner engagement while offering substantial evidence to support a shift in physical education classroom instruction that prioritizes student development.

## 2 Methods

### 2.1 Participants

Adolescents in China are divided into two categories: junior high school (Grades 7–9) and senior high school (Grades 10–12). Students in grades seven through 12 were the focus of this investigation. The sample size was determined by applying the rule of 10–15 cases per predictor, with 20 predictors necessitating 200–300 participants. A hybrid sampling strategy that integrates convenience sampling and random sampling was implemented: 10 junior high schools and six senior high schools in Chengdu and Chongqing were selected via convenience sampling. As key drivers of Western China's growth and the national Chengdu-Chongqing Economic Circle, Chengdu and Chongqing exhibit regionally representative school physical education development. Their distinct geographical and cultural characteristics provide an ideal setting for examining how teaching styles relationship student engagement in physical education, while their proximity enables diverse sampling. Random sampling: Two to three classes per grade were randomly selected within each school, and all students in these classes were invited to participate.

### 2.2 Data acquisition

**Figure 1:**
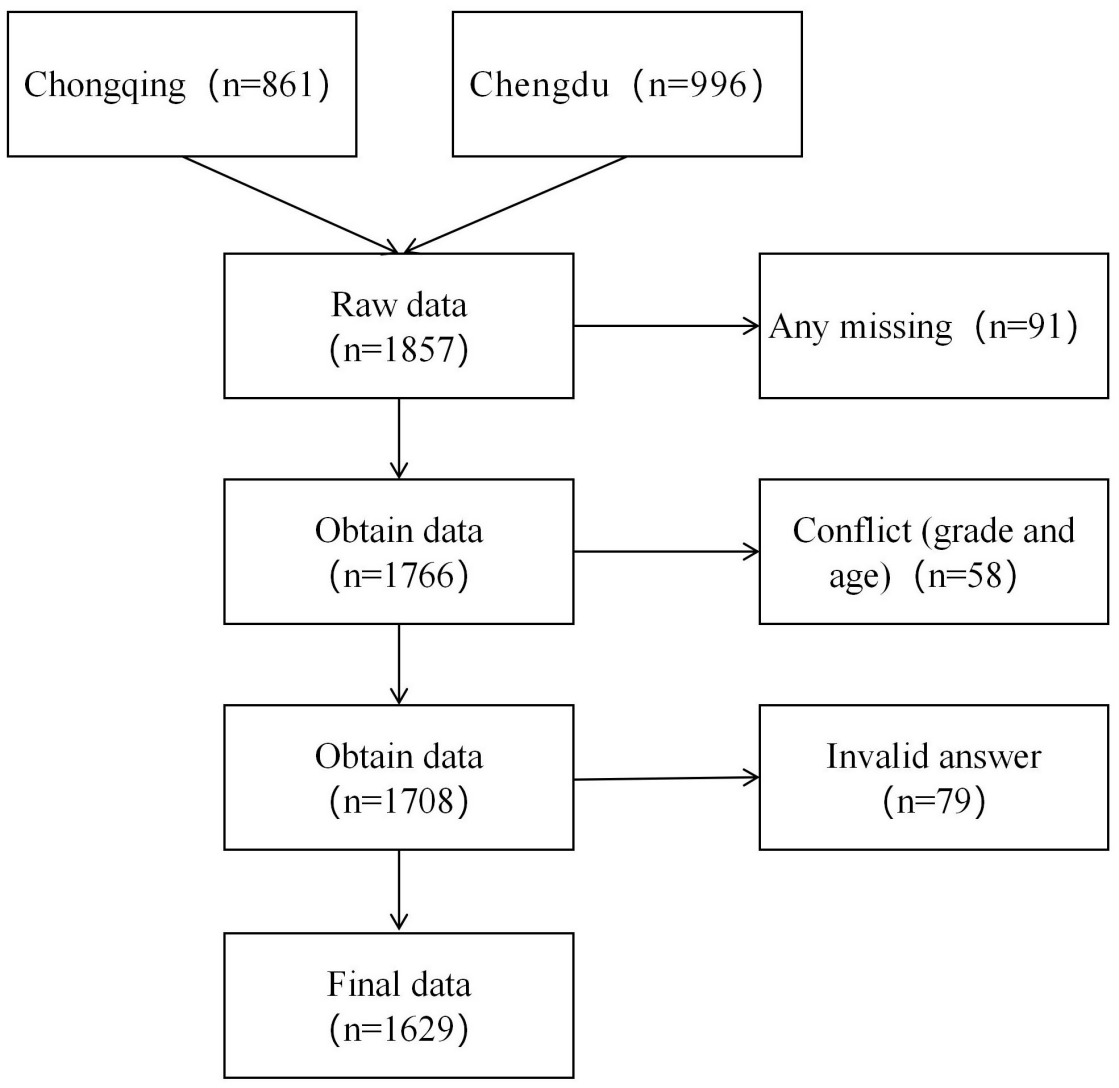
Participant screening process.

Between March and May 2025, Data were collected offline using Questionnaire Star, a Chinese online survey platform. Standardized data collection protocols were implemented to guarantee reliability and validity: (1) the study's purpose, voluntary participation, data utilization scope, and response guidelines were explained to students by researchers during the pre-survey briefing in order to reduce bias and increase engagement. (2) Survey administration: In order to guarantee independent and focused responses, questionnaires were completed in computer labs during physical education courses, with the guidance of a teacher and researcher. (3) Ethical compliance: The study was conducted in accordance with the institutional ethical guidelines and the Declaration of Helsinki. The Ethics Committee of the Shaanxi Vocational and Technical College endorsed the protocol. Informed assent was obtained from minors along with written parental consent: Student assent: Verbal consent from students. Parental consent: Written confirmation from parents/guardians through class-specific communication groups. (4) Data anonymity: No personally identifiable information was gathered. Throughout the storage and analysis processes, all data were anonymized and encrypted. (5) Data processing: To ensure the breadth and representativeness of the sample, a total of 1,857 questionnaires were distributed. During data collation, missing values and invalid answer were deleted from the sample questionnaires (Figure 1), resulting in a final valid sample of 1,629 (87.7% validity rate).

### 2.3 Instruments and variables

All measurement instruments utilized in this investigation were validated scales with established reliability and validity, which encompassed psychological resilience, classroom satisfaction, learning engagement, and teaching styles. Additionally, demographic queries that were self-designed were implemented to gather data regarding the age, gender, grade, height, and weight of adolescents.

#### 2.3.1 Scale for teaching style

He et al. (2011) developed the associationive Teaching Style Scale for Teachers to assess students' perceived PE teaching styles. This scale divides teaching styles into four dimensions: innovative-exploratory, rigorous-logical, humorous-active, and caring-sharing. It consists of 18 items that are rated on a Likert 7-point scale, with higher scores indicating a stronger alignment with a specific teaching style (e.g., the teacher employs humor and energy to engage students). The scale ranges from 1 (strongly disagree) to 7 (strongly agree). The scale has been extensively utilized in educational research and has exhibited exceptional reliability (Cronbach's α = 0.913) (Hong and Feng, 2022; Rongrong et al., 2017).

#### 2.3.2 Scale for sports learning engagement

The Chinese version of the Sports Learning Engagement Scale, which was adapted by Zhang et al. (2020) from the Utrecht Work Engagement Scale-Student (UWES-S) (Schaufeli et al., 2002), was employed to evaluate the sports learning engagement of adolescents. This 17-item scale is rated on a Likert 5-point scale, with 1 indicating strong disagreement and 5 indicating strong agreement. The three dimensions are vigor, dedication, and assimilation. Higher scores indicate a higher level of engagement. The scale demonstrated an exceptional level of reliability (Cronbach's α = 0.968).

#### 2.3.3 Classroom satisfaction scale

The classroom satisfaction of students was assessed using the Physical Education Class Satisfaction Scale, which was revised by Ma Y. (2024). This 35-item scale assesses five domains: teaching competence, facility provision, school support, learning outcomes, and classroom climate. It is rated on a Likert 5-point scale, with 1 indicating very dissatisfaction and 5 indicating very satisfaction. A higher score suggests that the individual is more satisfied. A high level of reliability was obtained by the scale (Cronbach's α = 0.959).

#### 2.3.4 CD-RISC psychological resilience scale

This study measured adolescent resilience using the validated Chinese version of the Connor-Davidson Resilience Scale (Connor and Davidson, 2003), culturally adapted and psychometrically validated by Yu and Zhang (2007). This 25-item scale is scored on a 5-point scale, with 0 representing strongly disagree and 4 representing strongly concur, and it encompasses three dimensions: tenacity, strength, and optimism. Higher scores indicate greater resilience. The scale has been extensively validated in Chinese contexts (Cai et al., 2018; Huang et al., 2024) and has exhibited strong reliability (Cronbach's α = 0.91). The basic information of all scales used in this study is shown in Table 1.

**Table 1:** Summary of measurement tools.

**Scale name**	**Measurement dimensions**	**Number of items**	**Likert**	**Cronbach's α**
Teaching styles	Humorous-active	6	1–7	0.913
	Rigorous-logical	5		
	Innovative-exploratory	4		
	Caring-sharing	3		
Sports learning engagement	Vigor engagement	6	1–5	0.968
	Dedication engagement	5		
	Absorption engagement	6		
Classroom satisfaction	Teaching competence	7	1–5	0.959
	Facility satisfaction	7		
	School support	9		
	Learning outcomes	6		
	Classroom climate	6		
Psychological resilience	Tenacity	13	0–4	0.91
	Strength	7		
	Optimism	5		

### 2.4 Statistical analysis

This study employed IBM SPSS Statistics 26.0 for statistical analysis and data processing. In the descriptive statistics, categorical variables were presented as frequencies and percentages, while continuous variables were reported as means and standard deviations. First, the normality of the main variables was confirmed through the Kolmogorov-Smirnov (K-S) test, Q-Q plots, and P-P plots, which supported the use of independent samples *t*-tests for group comparisons the figures for normality tests are shown in Supplementary material 1. Second, Harman's Single Factor Test was conducted to assess common method bias and evaluate potential systematic errors in the data. Third, Pearson correlation analysis was performed to examine the relationships among psychological resilience, classroom satisfaction, sports learning engagement, and teaching styles. Fourth, multiple linear regression analysis was used to test the predictive association of physical education teaching styles on learning engagement after controlling for confounding variables. During this process, the Variance Inflation Factor (VIF) was calculated to diagnose multicollinearity among independent variables, with thresholds set as follows: VIF = 1 indicated no multicollinearity, 1 < VIF < 5 suggested mild multicollinearity, and VIF ≥ 5 indicated severe multicollinearity. Finally, a parallel mediation model was constructed using the SPSS PROCESS Macro (v4.1) to test the indirect associations of psychological resilience and classroom satisfaction in the relationship between teaching styles and learning engagement. Bootstrap sampling (5,000 iterations with 95% confidence intervals) was used to test significance. The significance level for this study was set at α = 0.05.

## 3 Results

### 3.1 Common method bias test

In this study, data were collected using self-report methods, which may involve common method bias. Therefore, Harman's single-factor test was employed to examine common method bias. The results showed that there were eight factors with eigenvalues < 1, and the first factor explained only 49.1% (< 50%) of the variance (Podsakoff et al., 2003), indicating that common method bias did not significantly affect the research results.

### 3.2 Demographic characteristics of participants

The study consisted of 1,629 adolescents, with a mean age of 14.31 ± 2.29 years. The cohort was divided between 785 males (48.2%) and 844 females (51.8%). Among these, 1,178 (72.3%) were junior high school students (Grades 7–9) and 451 (27.7%) were senior high school students (Grades 10–12). Within the normal range, the mean BMI was 20.63 ± 4.42 kg/m^2^. Key variables that were assessed were as follows: the perceived PE instructional styles are relatively high (101.36 ± 26.05) (total possible score: 126). Level of engagement in sports learning (total possible score: 105): moderate (64.65 ± 17.93). Relatively high level of classroom satisfaction (total possible score: 175) (141.45 ± 32.99). Level of psychological resilience: moderate (70.73 ± 22.22) (maximum possible score: 100). The demographic and variable characteristics are presented in detail in Table 2.

**Table 2:** Demographic characteristics of participants.

**Continuous variables**	**Mean ±SD**	**Categorical variables**	**Frequency**	**Percentage (%)**
Age	14.31 ± 2.29	Gender	/	/
BMI	20.63 ± 4.42	Male	785	48.2
Teaching styles	101.36 ± 26.05	Female	844	51.8
Sports learning engagement	64.65 ± 17.93	Grade level	/	/
Classroom satisfaction	141.45 ± 32.99	Junior high school	1,178	72.3
Psychological resilience	70.73 ± 22.22	Senior high school	451	27.7

### 3.3 Group comparisons by gender and grade level

Male students reported significantly higher levels of physical education learning engagement than for female students (*P* < 0.05), while there was no statistically significant difference in perceived physical education teachers‘ teaching styles between genders (*t* = 1.321, *P* = 0.187). In addition, there were no statistical differences in perceived PE teachers' teaching styles, PE learning engagement, classroom satisfaction, and psychological resilience between grades (*p* > 0.05). There was no statistical difference in the four types of teaching styles of physical education teachers in HA, RL, IE, and CS as perceived by male and female students between genders and grades (*p* > 0.05).

In terms of physical education learning engagement, boys were significantly more engaged than girls in vigor engagement, dedication engagement, and concentration engagement (*p* < 0.05), whereas only concentration engagement differed significantly between grades, and was significantly higher in high school than in middle school (*t* = −4.054, *P* = 0.04).

In terms of classroom satisfaction, boys were significantly more satisfied than girls in terms of teaching ability, site equipment, school support, school associationiveness, and classroom climate (*p* < 0.05), while between grades only site equipment satisfaction was significantly higher in middle school than in high school (t = 2.650, P = 0.008).

In terms of psychological resilience, boys were significantly higher than girls in resilience, self-improvement, and optimism (*p* < 0.05), however, there was no statistically significant difference in the psychological resilience of students between grades (*p* > 0.05). The results of the comparative analysis between groups are detailed in Table 3 and Supplementary material 2.

**Table 3:** Group comparisons by gender and grade level.

**Variable**	**Male (*n* = 785)**	**Female (*n* = 844)**	***t***	***p***	**Cohen's d**	**Junior high (*n* = 1,178)**	**Senior high (*n* = 451)**	***t***	***p***	**Cohen's d**
Teaching styles	102.25 ± 26.74	100.54 ± 25.37	1.321	0.187	0.066	101.06 ± 25.65	102.15 ± 27.07	−0.756	0.450	0.041
Sports learning engagement	66.54 ± 11.70	62.89 ± 17.00	4.112	0.000	0.250	64.18 ± 18.43	65.88 ± 16.50	−1.801	0.072	0.097
Classroom satisfaction	144.5 ± 33.43	138.61 ± 32.34	3.611	0.000	0.179	141.99 ± 33.25	140.04 ± 32.29	1.066	0.286	0.060
Psychological resilience	73.52 ± 23.38	68.13 ± 20.76	4.905	0.000	0.244	70.71 ± 22.74	70.76 ± 20.82	−0.041	0.968	0.002

### 3.4 Correlations among perceived PE teaching styles, classroom satisfaction, psychological resilience, and sports learning engagement

Correlational analyses demonstrated substantial positive correlations between students' perceived PE teaching styles, classroom satisfaction, psychological resilience, and sports learning engagement (refer to Table 4). The following are the primary findings: classroom satisfaction (r = 0.573, *p* < 0.01), psychological resilience (r = 0.449, *p* < 0.01), and sports learning engagement (r = 0.543, *p* < 0.01) exhibited moderately strong positive correlations with perceived PE teaching styles. Classroom satisfaction was moderately positively correlated with psychological resilience (r = 0.445, p < 0.01) and sports learning engagement (r = 0.495, *p* < 0.01). A moderately strong positive correlation was observed between psychological resilience and sports learning engagement (r = 0.532, *p* < 0.01). In conclusion, the statistical significance and positive correlation of all pairwise relationships among perceived PE teaching styles, classroom satisfaction, sports learning engagement, and psychological resilience were both significant. In addition, there were significant positive correlations among the sub-dimensions of PE teachers' teaching styles, classroom satisfaction, psychological resilience, and sports learning engagement. The detailed results are shown in Supplementary material 3.

**Table 4:** Correlations among students' perceived PE teaching styles, classroom satisfaction, psychological resilience, and sports learning engagement.

**Variable**	**Teaching styles**	**Classroom satisfaction**	**Sports learning engagement**	**Psychological resilience**
Teaching styles	1			
Classroom satisfaction	0.574^*^	1		
Sports learning engagement	0.543^*^	0.495^**^	1	
Psychological resilience	0.449^*^	0.445^**^	0.532^**^	1

^*^p < 0.05, ^**^p < 0.01.

### 3.5 Association of PE teaching styles with students' sports learning engagement

In Table 5, the regression analysis results that investigate the correlation of students' perceived PE teaching styles and other variables on sports learning engagement are presented. Hypothesis H1 was thereby supported by the significant positive association with sports learning engagement that students' perceived PE teaching styles demonstrated after controlling for gender, grade, and BMI (β = 0.371, 95%CI = 0.343–0.398, *p* < 0.001). Subsequent examination of the four dimensions of teaching style disclosed distinct outcomes: the IE style demonstrated the strongest positive association (β = 0.898, 95%CI = 0.523–1.273, *p* < 0.001). A moderate positive association was observed for the RL style (β = 0.292, 95%CI = 0.066–0.518, *p* = 0.011). Engagement was significantly predicted by the CS style (β = 0.489, 95%CI = 0.179–0.800, *p* = 0.002). The HA style did not have a statistically significant correlation (β = 0.095, 95%CI = −0.092–0.282, *p* = 0.317). In addition, the VIF values of the independent variables all fell between 1.006 and 1.927, indicating that there was no multicollinearity. These results underscore the fact that IE, RL, and CS teaching styles are the primary factors that relationship sports learning engagement, whereas HA approaches lack significant predictive power.

**Table 5:** Regression analysis of students' perceived PE teaching styles for sports learning engagement.

**Variable**	**β**	**95%CI**	**Wald χ2**	***P***	**Variable**	**β**	**95%CI**	**Wald χ2**	***P***
Teaching styles	0.371	0.343, 0.398	682.033	0.000					
					HA	0.095	−0.092, 0.282	1.000	0.317
					RL	0.292	0.066, 0.518	6.426	0.011
					IE	0.489	0.179, 0.800	9.565	0.002
					CS	0.898	0.523, 1.273	22.031	0.000
Gender	3.234	1.777, 4.649	18.914	0.000		3.145	1.694, 4.597	18.036	0.000
Grade	0.581	−1.486, 2.649	0.304	0.582		0.723	−1.341, 2.786	0.471	0.492
BMI	0.014	−0.150, 0.178	0.028	0.868		0.013	−0.151, 0.177	0.024	0.876

HA, Humorous-Active; RL, Rigorous-Logical; CS, Caring-Sharing; IE, Innovative-Exploratory.

### 3.6 Mediating associations of classroom satisfaction and psychological resilience

**Figure 2:**
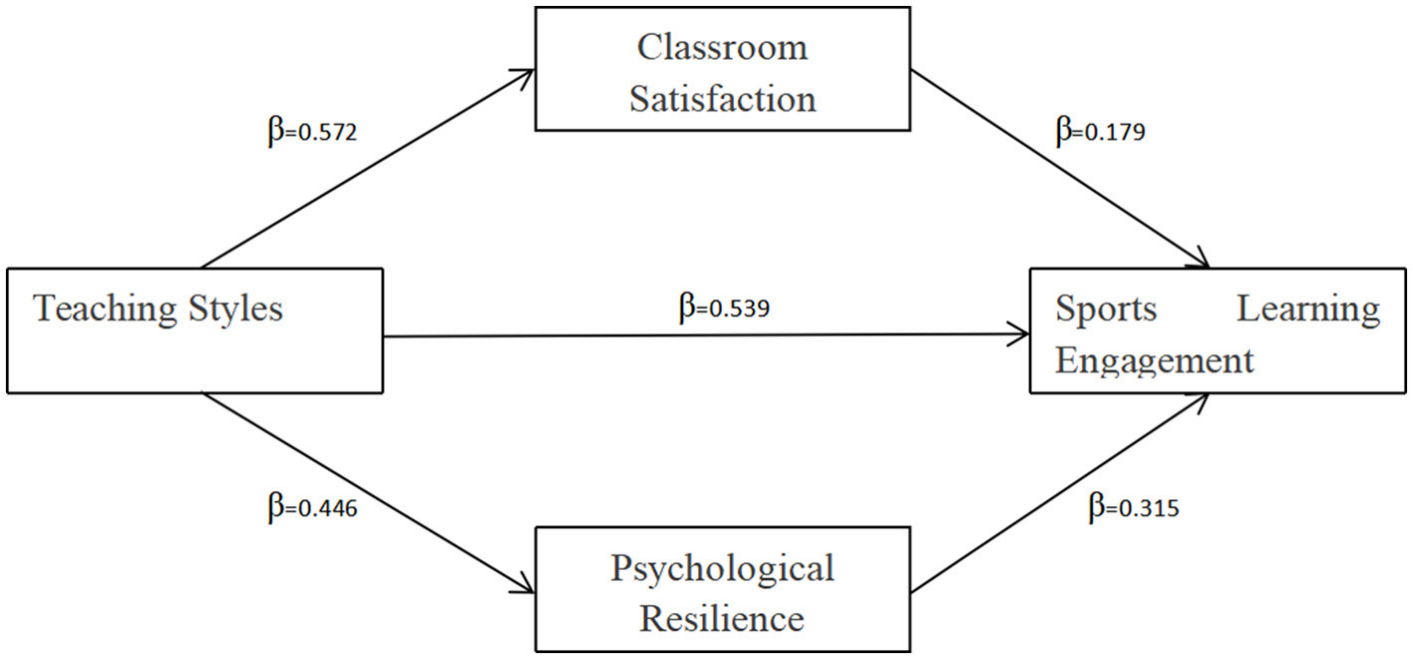
Mediation pathways of classroom satisfaction and psychological resilience between teaching styles and sports learning engagement.

To investigate the mediating roles of classroom satisfaction and psychological resilience in the relationship between PE teaching styles and sports learning engagement, a parallel mediation model was tested using the SPSS PROCESS Macro. Results (refer to Table 6) illustrated the following: classroom satisfaction: The indirect effect via classroom satisfaction in the association between teaching styles and engagement was significant (β = 0.189, 95%CI = 0.154–0.227, *p* < 0.05), accounting for 18.9% of the total association. Psychological Resilience: The indirect effect via psychological resilience in the association between teaching styles and engagement was also significant (β = 0.260, 95%CI = 0.215–0.308, *p* < 0.05), accounting for 26.0% of the total result. The direct association between teaching styles and engagement remained significant after accounting for mediators (β = 0.291, 95%CI = 0.254–0.329, *p* < 0.05). The partial mediation of both classroom satisfaction and psychological resilience is confirmed by these findings, which corroborate Hypotheses H2 and H3 (refer to Figure 2).

**Table 6:** Mediation analysis of classroom satisfaction and psychological resilience.

**Pathways**	**B**	**BootLLCI**	**BootULCI**	**Boot SE**	***p***	**β**	**Effect proportions**
Teaching styles = >classroom satisfaction = > sports learning engagement	0.070	0.069	0.140	0.018	0.000	0.102	18.9%
Teaching styles = >psychological resilience = > sports learning engagement	0.096	0.111	0.170	0.015	0.000	1.405	26.0%
Teaching styles = >>classroom satisfaction	0.724	0.674	0.774	0.026	0.000	0.572	
Classroom satisfaction = >sports learning engagement	0.097	0.072	0.122	0.013	0.000	0.179	
Teaching styles = >sports learning engagement	0.204	0.172	0.236	0.016	0.000	0.539	
Teaching styles = >sports learning engagement	0.371	0.343	0.399	0.014	0.000	0.539	
Teaching styles = >psychological resilience	0.380	0.343	0.417	0.019	0.000	0.446	
Psychological resilience = >sports learning engagement	0.254	0.219	0.288	0.018	0.000	0.315	

## 4 Discussion

### 4.1 Key findings: significant positive correlation of students' perceived PE teaching styles on sports learning engagement

The results confirm a significant positive association between students' perceived PE teaching styles and their engagement (β = 0.371, *p* < 0.01), thereby validating Hypothesis H1. This finding aligns with self-determination theory (Ryan and Deci, 2017) and prior empirical studies (Leenknecht et al., 2017; Xiong, 2025; Yang and Dong, 2017), which propose that autonomy-supportive, structured, and emotionally nurturing teaching practices are associated with meeting students' psychological and developmental requirements and higher intrinsic motivation and behavioral engagement.

Analysis of Teaching Styles on a Subdimensional Scale: RL Style: By emphasizing structured lesson design and orderly activity organization, teachers mitigate instructional ambiguity, which in turn is associated with enhanced students' absorption engagement by providing predictable and coherent learning experiences (Martin, 2008).

CS Style: This style is associated with improved students' psychological safety and sustained engagement by fostering inclusive classroom climates, fostering emotional bonds between teachers and students, and providing emotional support (He et al., 2011; Shen et al., 2024). IE Style: The incorporation of innovative sports equipment, cutting-edge pedagogical tools, and novel activities contributes to proactive inquiry, creativity, and curiosity, thereby converting students from passive recipients to active learners (Ridwan et al., 2019). HA Style: Although humor can captivate students, Overreliance on entertainment (e.g., frequent quips, exaggerated gestures, or activities that deviate from instructional content) might compromise skill mastery. The positive correlation of these styles may be further diminished by adolescents' rising expectations for classroom professionalism, which may be perceived as unserious or inefficient (Bieg and Dresel, 2014; Bieg et al., 2019; Wanzer et al., 2010).

### 4.2 Mediation interpretation: mediating roles of classroom satisfaction and psychological resilience

This investigation verified the partial mediating roles of psychological resilience and classroom contentment in the association between perceived PE teaching styles and sports learning engagement, supporting Hypotheses H2 and H3. To be more precise, classroom satisfaction mediated 18.9% of the total association (indirect association = 0.070) (indirect association = 0.096). Psychological resilience mediated 26.0% of the total association. These findings are consistent with previous research conducted by Betoret (2007) and Fletcher and Sarkar (2016), which suggests that teaching styles indirectly increase engagement by promoting positive classroom environments (via satisfaction) and resilience-building (via psychological adaptability).

#### 4.2.1 A Subdimensional Analysis of Teaching Styles

Classroom satisfaction (association = 0.068) and psychological resilience (association = 0.162) demonstrate fully mediated associations in the RL style. Mechanism: Students' confidence in instructional competence is increased by structured teaching, which correlates with higher satisfaction (Martin, 2008). Additionally, anxiety is mitigated by the establishment of predictable challenges through explicit goal-setting and activity design (Fletcher and Sarkar, 2016). The innovative-exploratory style involves partial mediation through psychological resilience (association = 0.260) and classroom satisfaction (association = 0.175).

#### 4.2.2 Mechanism

Novel activities and tools are associated with provoked curiosity (Ma, 2024), while controlled risk-taking is linked to the development of resilience through iterative experimentation (González-Cutre et al., 2025). Resilience pathway: *p* > 0.05; association = 0.111; CS Style: Significant mediation only through classroom satisfaction. Resilience development necessitates extended exposure to supportive environments, while emotional support directly enhances teacher-student bonds and satisfaction (Curran and Standage, 2017; Sparks et al., 2015). HA Style: The absence of substantial mediation pathways is likely due to the fact that adolescents' expectations for instructional professionalism are often in conflict with mismatched humor (Bieg et al., 2019). It is noteworthy that the mediating role of psychological resilience exhibits stylistic differences. The IE style activates psychological resilience by creating challenging learning tasks, allowing students to engage in trial-and-error and overcome obstacles (Masten, 2014), thereby enhancing self-efficacy. In contrast, the CS style emphasizes emotional support and fulfills the need for belonging, with its associations directly reflected in classroom satisfaction. Psychological resilience typically develops over time (Ungar, 2015), and the cross-sectional design of this study may not have captured its dynamic process. Furthermore, while the caring-sharing style provides “protective factors”, these may not necessarily translate directly into resilience (Doan et al., 2022). Future research should adopt longitudinal designs to examine the long-term associations of the caring-sharing style on psychological resilience.

Furthermore, the mediating role of psychological resilience (accounting for 26.0% of the total association) was significantly stronger than that of classroom satisfaction (18.9%). This difference is primarily attributable to the following factors: On one hand, psychological resilience, conceptualized as a stable internal trait (Connor and Davidson, 2003), operates through a deeper and more enduring mechanism. In this study, the innovative and logically rigorous teaching style, characterized by the design of challenging tasks, structured support, and emotional care, associationively fostered students' psychological resilience when encountering difficulties in physical education (PE) learning. The enhancement of this capacity helps regulate negative emotions (Gross, 2015), sustain higher levels of self-efficacy, and thereby facilitate sustained learning engagement in PE. In contrast, classroom satisfaction reflects students' immediate affective experiences and evaluations within the specific context of PE classroom instruction (Guolla, 1999). While it exerts a positive relationship on learning engagement to some extent, it is more susceptible to contextual fluctuations such as student mood swings, variations in facility arrangements, changes in task difficulty, and the quality of teacher-student interactions (Betoret, 2007; Moore and Fry, 2017). Consequently, its correlation tends to be more focused on enhancing participation willingness within individual class sessions (Diloy-Peña et al., 2021). On the other hand, the inherent characteristics of the PE discipline amplify the role of psychological resilience. PE learning inherently involves high-pressure situations such as physical exertion, public performance, and competitive encounters, which continuously demand the mobilization of psychological endurance and resilience (Galli and Gonzalez, 2015). Although a highly satisfying classroom atmosphere can mitigate negative experiences, it does not eliminate the inherent challenges of the PE learning process itself. Therefore, the pathway driving learning engagement through psychological resilience emerges as more robust (Trigueros and García-Mas, 2025).

### 4.3 Cultural implication

This study reveals that physical education (PE) teaching in China needs to adhere to the principle of “teaching through rigor” (Jin and Cortazzi, 2006). PE teachers humor and liveliness should serve instructional objectives, content, and skill acquisition, rather than merely enlivening the classroom atmosphere. Relationship by traditional Chinese culture, classroom instruction emphasizes teacher authority, and classroom discipline (Li and Wegerif, 2014). Consequently, students expect PE teachers to demonstrate professionalism over entertainment. The Confucian concept of being “strict yet caring” helps explain why the “caring-sharing” and “rigorous-logic” teaching styles prove significantly more associationive. The former resonates with the “benevolence” ideal in teacher-student relationships, while the latter aligns with the role responsibility of “imparting knowledge and moral guidance”. Conversely, humor and liveliness disconnected from teaching objectives and content can easily be perceived by students as “inassociationive talk” and “undermining learning seriousness” (Van Praag et al., 2017). This is particularly true in PE, a subject primarily focused on sports skills instruction, where students prioritize the process and experience of learning motor techniques over their teachers' entertaining behaviors. This phenomenon stands in sharp contrast to findings in Western contexts where “humor fosters classroom relaxation” (Bieg and Dresel, 2018; Fredrickson, 2001), further confirming the cultural heterogeneity in teaching style associationiveness (Nisbett, 2010). Therefore, future reforms in Chinese PE should prioritize developing teachers' structured innovation and emotionally immersive care.

### 4.4 Gender and grade differences

Our study also revealed significant gender differences in physical education engagement and psychological resilience, with males scoring significantly higher than females (*p* < 0.01). This disparity may stem from gender socialization's relationship on sports participation (Chalabaev et al., 2009). Males are often encouraged toward high-intensity competitive sports, while females frequently encounter “negative stereotypes about athletic ability” (Avraam and Anagnostou, 2022) and body image anxiety, reducing their engagement. Therefore, physical education teachers could implement gender-specific activities, and schools might develop differentiated curricula to better safeguard students' rights to enjoy sports.

Regarding grade levels, high school students demonstrated significantly greater focused engagement (*p* < 0.05) but lower satisfaction with facilities and equipment (*p* < 0.01). This reflects high schoolers' prioritization of physical education's “stress-relief function” under China's academic pressure (Liu, 2025), coupled with institutional neglect of sports resources. Conversely, interest-driven middle schoolers exhibited greater tolerance for facility limitations (Eccles and Roeser, 2011). We recommend targeted interventions: enhancing psychological resilience training for high school students while ensuring adequate equipment allocation in middle schools to cater to students' interests and needs.

### 4.5 Implications for teacher training

This research contributes to the body of literature in educational psychology and physical education by delineating the correlations between teaching styles and the engagement of students in sports learning. It provides innovative insights for the optimization of pedagogical practices. The results have practical implications for instructional design and teacher development: (1) Improvements to Teacher Training: Emphasize the development of competencies in the areas of IE, RL, and CS. For example, promote the integration of evidence-based pedagogical methods and emerging technologies. Enhance the proficiency of educators in the development of structured lesson plans and the instruction of sport-specific skills. (2) Classroom Satisfaction Optimization: Promote autonomy by providing real-time feedback (e.g., wearable devices for performance monitoring) while ensuring safe and adequate sports facilities. Boost participation by implementing student-centered activities, such as peer-led skill demonstrations. (3) Resilience-Building Interventions: Combine attribution retraining with adversity training modules to reframe setbacks as growth opportunities. (4) Gender-Sensitive Pedagogy: By implementing elective-based modular teaching, the engagement disparity between genders can be bridged, enabling students to select activities that are in accordance with their interests.

### 4.6 Limitations and future work

The study has limitations, despite the fact that the hypotheses were confirmed: (1) Methodological Constraints: The use of self-report questionnaires and the cross-sectional design limit the ability to infer causal relationships and may neglect contextual factors and nuanced motivations. Qualitative methods, such as classroom observations and interviews, should be employed to triangulate the results of future research. (2) Cross-sectional design: the inability to establish causality necessitates longitudinal or experimental designs (e.g., monitoring engagement changes over a semester under varying teaching styles). (3) The samples in this study were all from the Chinese region, and it is possible that specific cultures may relationship students' perceptions of and responses to teaching styles, and the generalizability of its findings to other cultural contexts needs to be verified. Future research should employ longitudinal or experimental designs to better examine potential causal links, for example, by monitoring engagement changes over time in conjunction with varying teaching styles.

## 5 Conclusion

This cross-sectional study, involving 1,629 Chinese adolescents, investigated the correlation mechanisms of physical education (PE) teachers' teaching styles on students' engagement in PE learning, while examining the mediating roles of class satisfaction and psychological resilience. Key findings indicate that the TE (β = 0.898), RL (β = 0.292), and CS (β = 0.489) styles showed significant positive associations with learning engagement. Conversely, the HA style showed no significant association (β = 0.095, *p* = 0.317), suggesting Chinese students' prioritization of professionalism in PE instruction within their cultural context.

Mediation analyses revealed that both classroom satisfaction and psychological resilience partially mediated the relationship between teaching style and learning engagement (*p* < 0.05), with the role of psychological resilience (26.0%) being significantly stronger than that of classroom satisfaction (18.9%). This difference highlights the distinctive nature of physical education. By incorporating specifically designed challenging tasks, structured scaffolding support, and emotional care, it associational enhances students' psychological resilience when facing difficulties in PE learning. This approach plays a pivotal role in achieving sustained learning engagement. At the group level: males reported significantly higher PE learning engagement and psychological resilience than females (*p* < 0.01), highlighting the relationship of gender socialization on sports participation; high school students exhibited greater focused engagement but lower satisfaction with facilities (*p* < 0.01), reflecting resource allocation imbalances and neglect of PE amidst academic pressure. Therefore, to promote student engagement in PE learning, it is recommended to prioritize the development of teachers' innovative exploration, rigorous logic, and caring sharing competencies, optimize PE curricula, improve guarantees for school equipment, and strengthens psychological resilience training. Limitations include the cross-sectional design and reliance on self-reported data. Future research should employ longitudinal or experimental data to verify the long-term associations of teaching styles, conduct cross-cultural comparisons to enhance generalizability, and integrate multiple data sources (e.g., classroom observations, physiological indicators) to improve explanatory power.

## Data Availability

The raw data supporting the conclusions of this article will be made available by the authors, without undue reservation.
