# Supplementary figures and images for: Teaching styles and sports engagement: mediation by satisfaction and resilience in Chinese adolescents
Source: Front Psychol. 2025 Jul 7;16:1630300. doi: 10.3389/fpsyg.2025.1630300 (PMC12277347; doi:10.3389/fpsyg.2025.1630300)

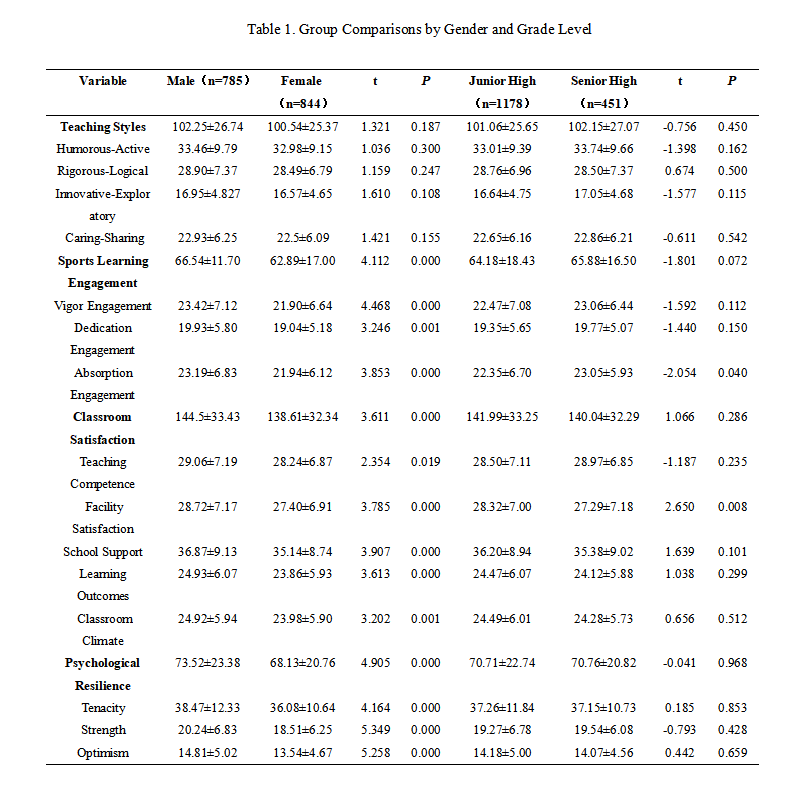

Supplement: Supplementary file 1 [file Supplementary_file_1.png]

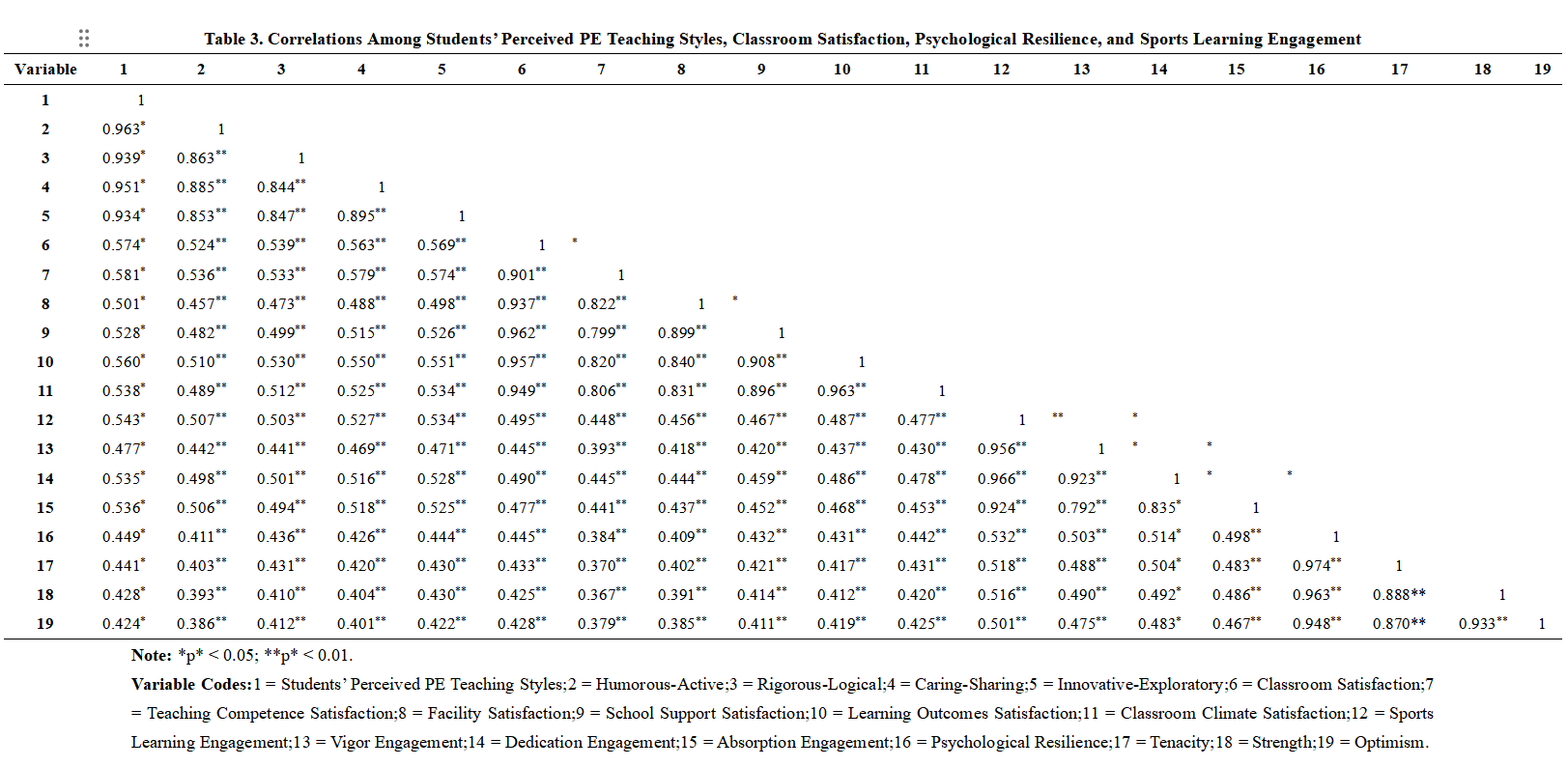

Supplement: Supplementary file 2 [file Supplementary_file_2.png]
