# Supplementary material for: Teaching styles and sports engagement: mediation by satisfaction and resilience in Chinese adolescents
Source: Front Psychol. 2025 Jul 7;16:1630300. doi: 10.3389/fpsyg.2025.1630300 (PMC12277347; doi:10.3389/fpsyg.2025.1630300)
Supplement: Supplementary file 4 [file Data_Sheet_1.pdf]

Q-Q图

Teaching Styles

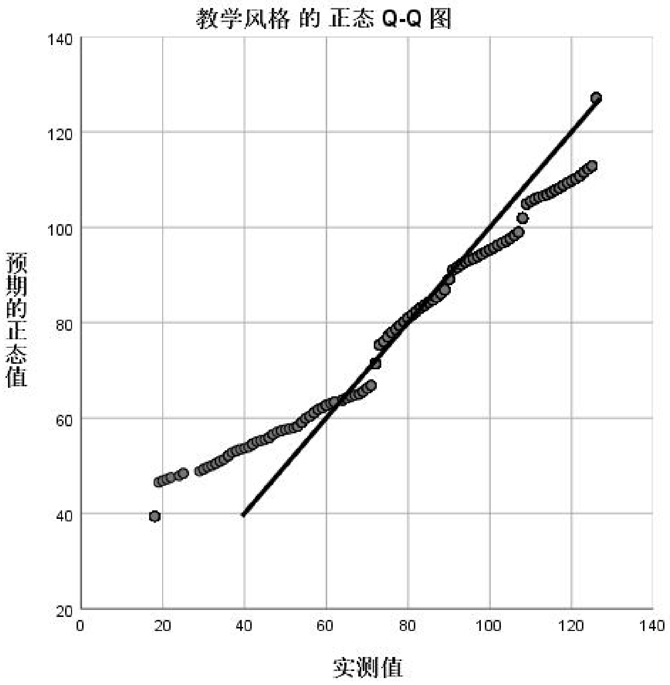

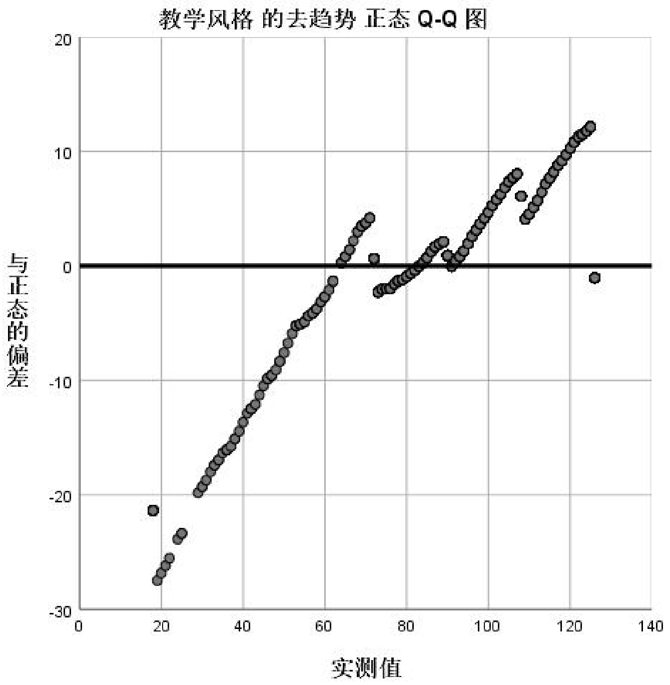

Classroom Satisfaction

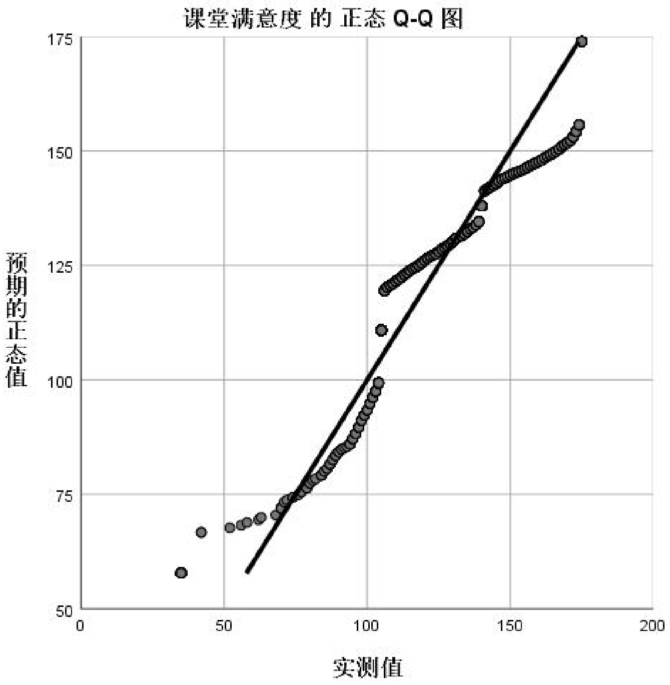

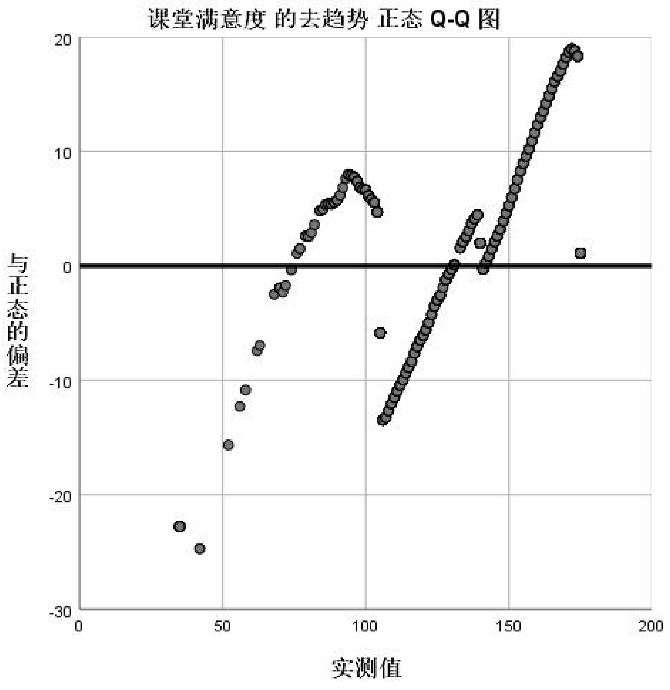

Sports Learning Engagement

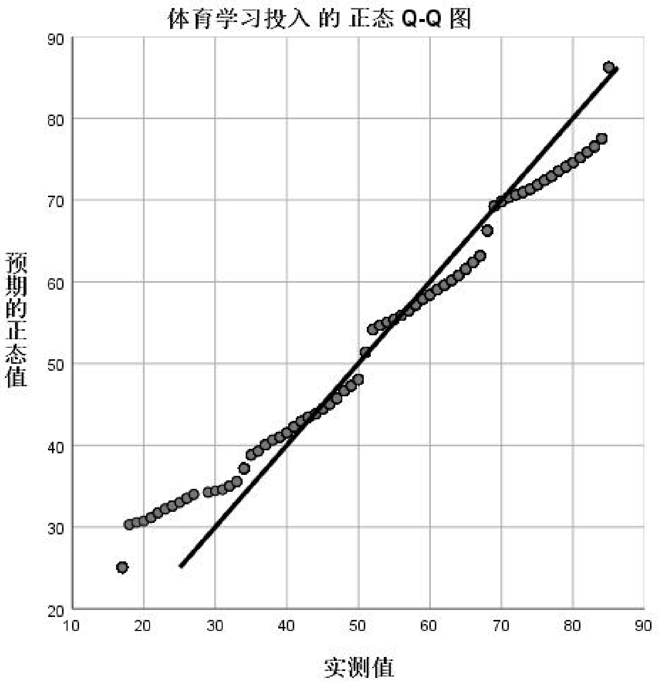

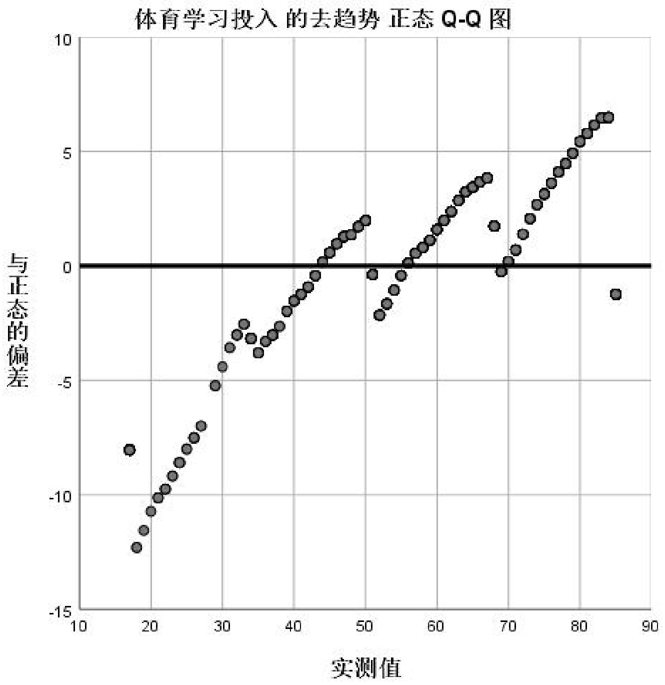

Psychological Resilience

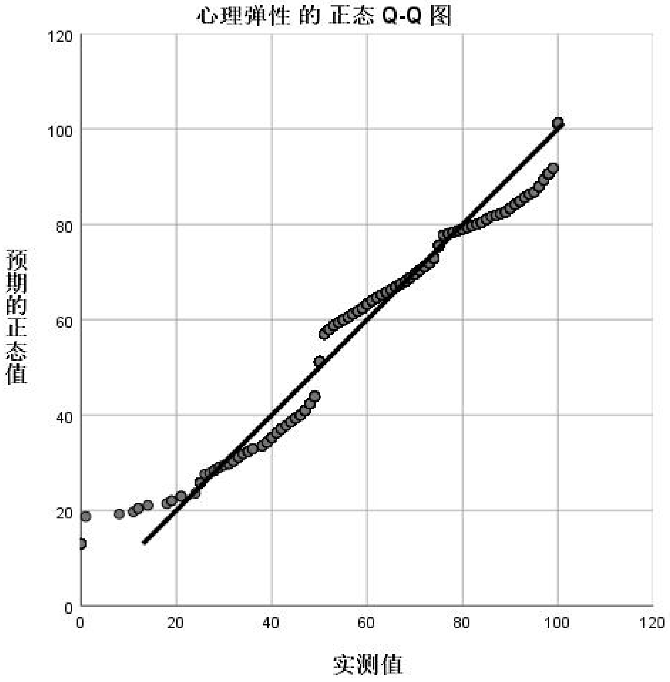

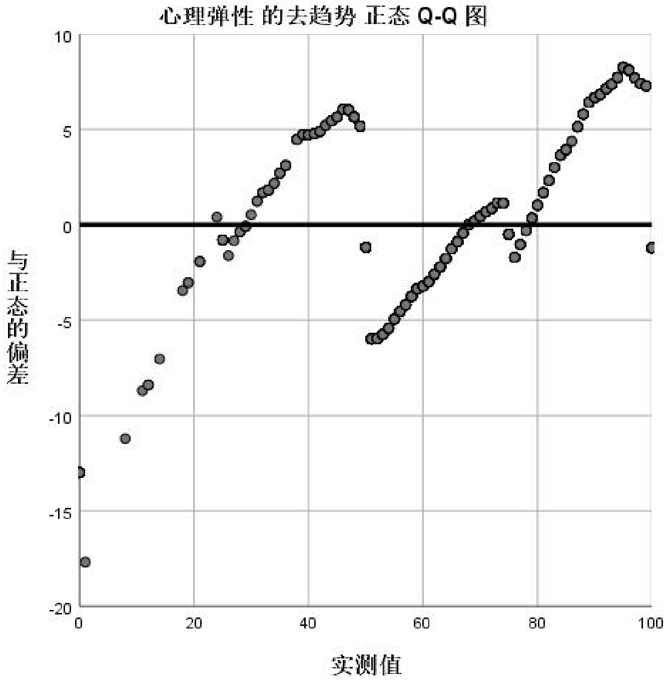

P-P图

Teaching Styles

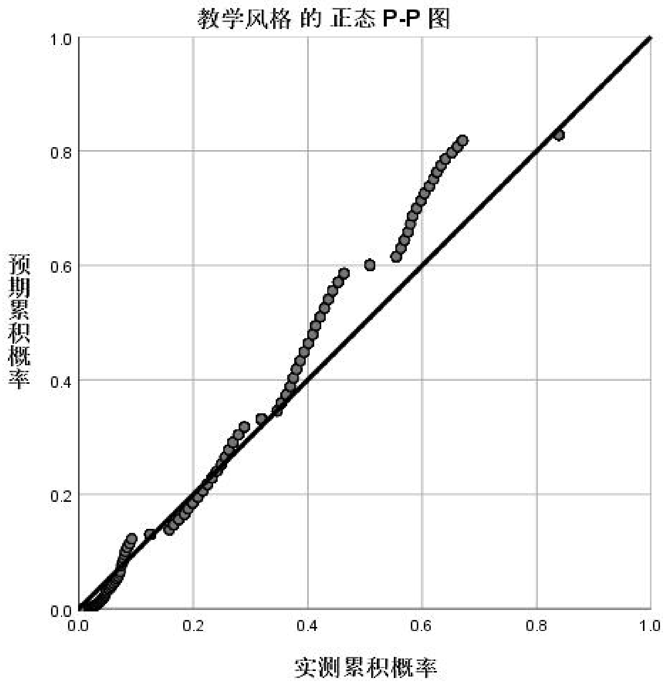

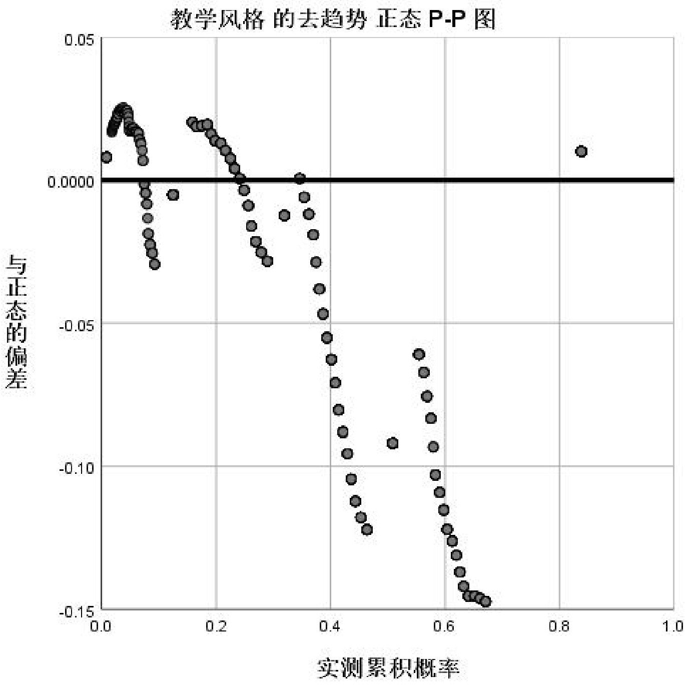

Classroom Satisfaction

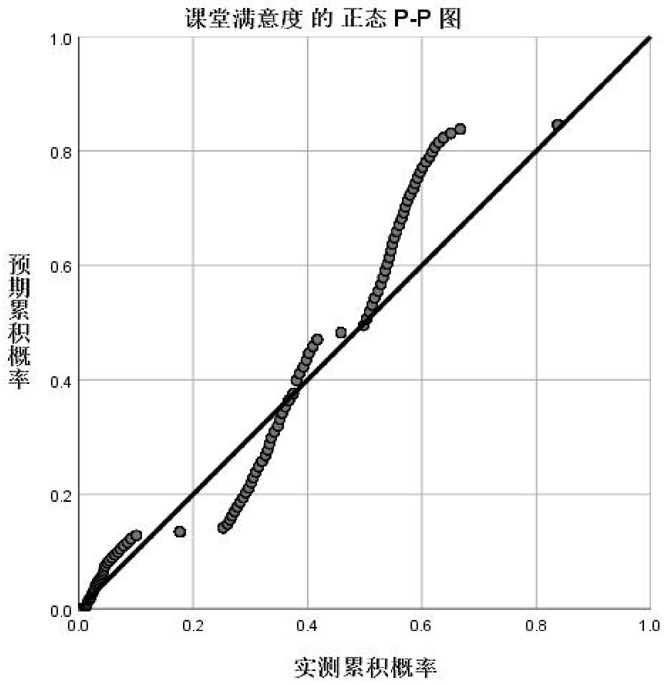

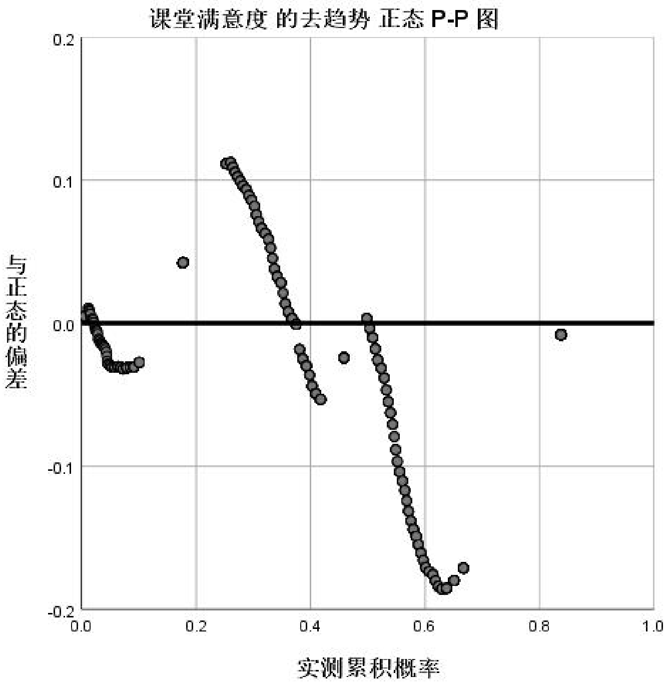

Sports Learning Engagement

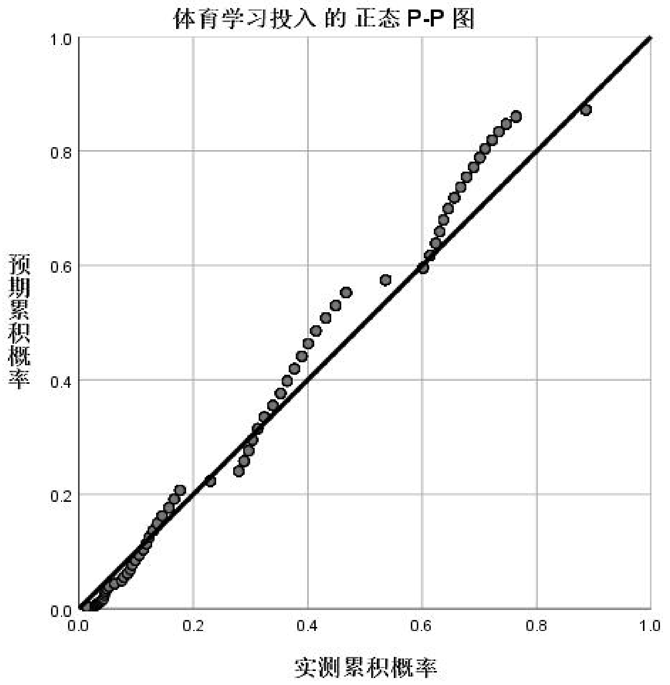

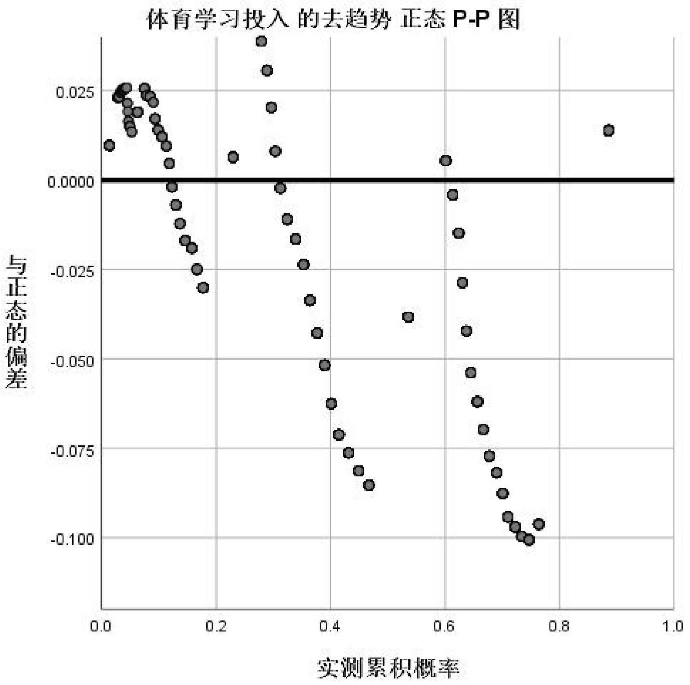

Psychological Resilience

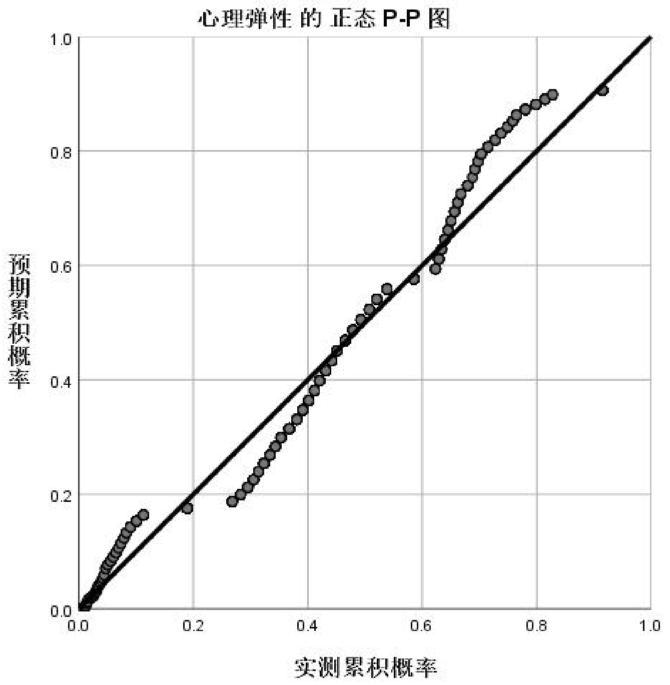

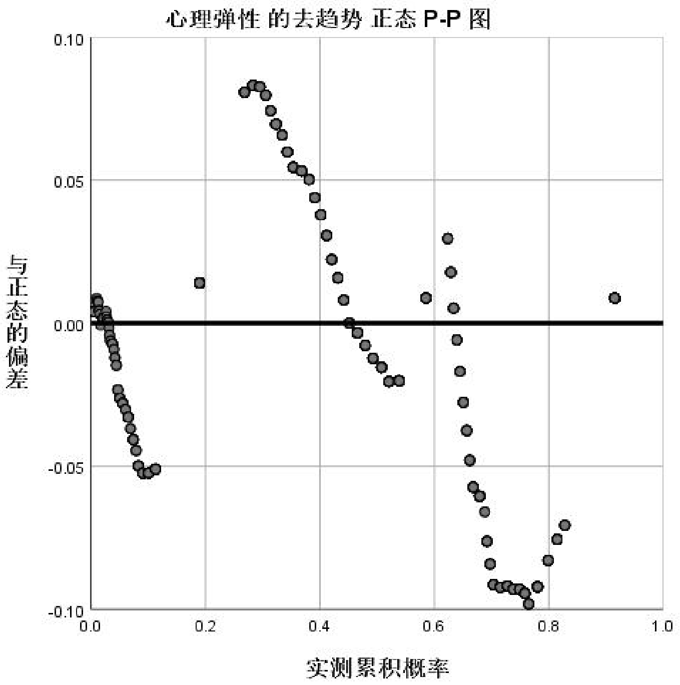

K-S Test

variable	statistics	Df	P
Teaching Styles	0.172	1629	0
Classroom Satisfaction	0.19	1629	0
Sports Learning Engagement	0.128	1629	0
Psychological Resilience	0.106	1629	0
